# ﻿A new species of the genus *Soriculus* (Soricidae, Eulipotyphla, Mammalia) from Medog in the eastern Himalaya

**DOI:** 10.3897/zookeys.1195.115699

**Published:** 2024-03-14

**Authors:** Xiaoxin Pei, Zhongzheng Chen, Quan Li, Xueyou Li, Changzhe Pu, Kang Luo, Jing Luo, Mingjin Pu, Hongjiao Wang, Laxman Khanal, Xuelong Jiang

**Affiliations:** 1 State Key Laboratory of Genetic Resources and Evolution & Yunnan Key Laboratory of Biodiversity and Ecological Security of Gaoligong Mountain, Kunming Institute of Zoology, Chinese Academy of Sciences, Kunming, China Kunming Institute of Zoology, Chinese Academy of Sciences Kunming China; 2 Kunming College of Life Sciences, University of Chinese Academy of Sciences, Kunming, China University of Chinese Academy of Sciences Kunming China; 3 Collaborative Innovation Center of Recovery and Reconstruction of Degraded Ecosystem in Wanjiang Basin Co-founded by Anhui Province and Ministry of Education, Anhui Normal University, Wuhu, China Anhui Normal University Wuhu China; 4 Central Department of Zoology, Institute of Science and Technology, Tribhuvan University, Kathmandu, Nepal Tribhuvan University Kathmandu Nepal

**Keywords:** Phylogeny, small mammals, taxonomy

## Abstract

Himalayan shrews of the genus *Soriculus* (Soricidae, Eulipotyphla), currently represented by four nominal species, are endemic to the Himalayas and the Gaoligong Mountains. In April 2022 and April 2023, a total of 10 specimens of *Soriculus* were collected from Beibeng and Damu, Medog County, Tibet, China. The morphology of the specimens was compared with the four recognised species of the genus *Soriculus*. Additionally, two mitochondrial (*Cyt b* and *12S*) and three nuclear (*APOB*, *BRCAI* and *RAG2*) genes were sequenced to test the phylogenetic relationships of these specimens with the other species. Our results indicate that these specimens represent a distinct species, *Soriculusbeibengensis***sp. nov.**, which is formally described here. The new species is distinguished from the other *Soriculus* species by the combination of darker pelages, smaller size, the relatively stubby nasal and the widened posterior processes of incisors. Phylogenetic analyses revealed the new species is sister to *S.minor*. The p–distance of *Cyt b* gene between *S.beibengensis* sp. nov. and other nominal *Soriculus* species ranges from 9.1–16.3%. This new species has a known distribution at an elevation of 1,500–2,125 m in Medog County, Tibet, China. The discovery of this new species from Medog County has important implications for interpreting small mammal biogeographic patterns in the eastern Himalaya and the mountain chains of south-west China.

## ﻿Introduction

The genus *Soriculus* Blyth, 1854 (Mammalia, Eulipotyphla, Soricidae) is an endemic genus in the Himalayas and Gaoligong Mountains and which is mainly distributed in countries and regions across the eastern Himalayas (Bhutan, Sikkim of India, Nepal, and Yunnan and Tibet of PR China) (Hutterer 2005; Motokawa et al. 2008; Li et al. 2024). The small size and preference to inhabit remote forested mountainous areas make it challenging to collect specimens, limiting further studies.

For a long time, *Episoriculus* Ellerman and Morrison-Scott, 1966 and *Chodsigoa* Kastchenko, 1907 were considered subgenera or junior synonyms of the genus *Soriculus* (Ellerman and Morrison-Scott 1951; Hoffmann 1986; Corbet 1992). However, Repenning (1967) distinguished them into three separate genera, based on the mandibular and dental characteristics, which have since become widely recognised (Hutterer 2005). The first described species of the genus *Soriculus* was *Soriculusnigrescens* (Gray, 1842) from the specimens collected in West Bengal, India. For many decades, the genus was known to include only this species and two subspecies (*S.n.nigrescens* and *S.n.minor* Dobson, 1890) (Burgin and He 2018). Jiang et al. (2023) revealed deep divergence within the genus *Soriculus* and proposed *S.n.minor* should recover the full species status and might represent a new genus. Recently, Chen et al. (2023) systematically reviewed the taxonomy of the genus and described two new species *Soriculusnivatus* (Chen & Jiang, 2023) and *Soriculusmedogensis* (Chen & Jiang, 2023) from the eastern Himalayas and elevated *S.n.minor* as a distinct species (*S.minor*). Meanwhile, Chen et al. (2023) reported another putative species (referring to *Soriculus* sp. 3 in their study) in Medog. However, because only one specimen was collected, this specimen was not described systematically.

Medog is located in the eastern Himalayas with complex climate and geographic structure, making a biodiversity rich region. Due to the remoteness of Medog and the limited field surveys in the area, the biodiversity has remained poorly known and underestimated. Multiple new taxa have been recently described from the area, including mammals, for example, one new genus and species of mole (Chen et al. 2021), three new mountain voles (Liu et al. 2022) and two new shrews (Chen et al. 2023). In April 2023, we conducted further field surveys in Medog and collected another 9 specimens of the putative species mentioned by Chen et al. (2023). Based on morphological and molecular phylogenetic analyses from multiple mitochondrial and nuclear loci, we confirmed that these specimens represent a distinct species of the genus *Soriculus*, which we describe herein as *Soriculusbeibengensis* sp. nov.

## ﻿Materials and methods

**Figure 1.**
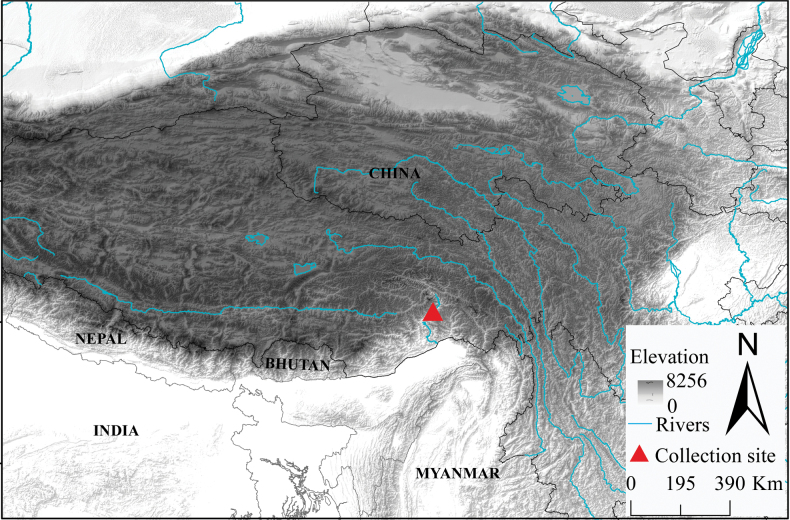
Map showing the collection sites of *S.beibengensis* sp. nov. in Beibeng, Medog, Tibet, China.

A total of 10 *Soriculus* specimens of *S.beibengensis* sp. nov. were collected from Medog, Tibet, China in April 2022 (Chen et al. 2023) and April 2023 (Fig. 1). Animals were handled, based on the animal care and use guidelines of the American Society of Mammologists (Sikes and the Animal Care and Use Committee of the American Society of Mammalogists 2016) and following the guidelines and regulations approved by the internal review board of the Kunming Institute of Zoology (KIZ), and with the permission of local government authorities. All specimens were deposited at the Kunming Natural History Museum of Zoology, Kunming Institute of Zoology (KIZ), Chinese Academy of Sciences, Kunming, China.

Five external measurements: weight (**W**), ear length (**EL**), head and body length (**HBL**), hind foot length (**HL**) and tail length (**TL**) were measured in the field and were reported to the nearest 0.1 g (for weight) or 0.5 mm (for different length categories). Eighteen craniodental metrics were measured using a digital caliper graduated to 0.01 mm following Chen et al. (2023). These metrics included (Table 1): basal length (**BL**), cranial breadth (**CB**), cranial height (**CH**), condyle-incisive length (**CIL**), distance between infra-orbital foramina (**DIF**), height of articular condyle (**HAC**), height of coronoid process (**HCP**), height of coronoid valley (**HCV**), interorbital breadth (**IOB**), lower tooth-row length (**LTR**), maxillary breadth (**MB**), mandibular length (**ML**), maximum width across the upper second molars (**M^2^M^2^**), distance from the front of upper fourth premolar to the end of upper third molar (**P^4^M^3^**), palato-incisive length (**PIL**), postpalatal depth (PPD), foramen magnum breadth (**BMF**) and upper tooth-row length (**UTL**). All craniodental measurements were taken by the single researcher (**ZZC**).

**Table 1.** Summary statistics (mean, standard deviation, ranges and number of samples) of external and skull measurements (in millimetres) of *Soriculus* specimens used in the study; character abbreviations are detailed in the “Material and methods”.

	*S.beibengensis* sp. nov.	*S.minor*	*S.nigrescens*	*S.medogensis*	*S.nivatus*
W	11.7 ± 1.4	8.9 ± 1.2	17.6 ± 2.5	13.3 ± 0.7	11.7 ± 1.7
	8.8–13.2; 10	7.7–12.1; 21	12.9–20.7; 11	12–14.1; 7	9.6–15.3; 23
HBL	77.2 ± 4.1	71 ± 3.9	88.6 ± 3.4	84.7 ± 0.8	82.6 ± 4.1
	70–81; 10	62–77; 20	83–93; 11	83–85; 7	70–90; 23
TL	40.6 ± 1.8	36.7 ± 4.1	45.8 ± 3.2	50.7 ± 3.6	51.6 ± 2.7
	38–44; 10	31.5–43; 21	42–52; 11	43–54; 7	46–58; 23
HF	12.6 ± 1.1	12.3 ± 0.6	15.4 ± 0.9	14.9 ± 0.7	15.3 ± 1
	11–14; 10	11–13.5; 21	14–17; 11	14–16; 7	13–17; 23
EL	8.1 ± 1.4	7.9 ± 1.1	8.7 ± 0.9	10.3 ± 1	9.3 ± 1.2
	6–10; 10	6–10; 6	8–11; 11	9–12; 7	7–12; 23
CIL	20.8 ± 0.3	19.6 ± 0.4	23.4 ± 0.5	23.7 ± 0.7	23.4 ± 0.3
	20.4–21.3; 10	19.2–20.2; 7	22.5–24.2; 9	22.7–24.5; 4	22.8–24.1; 22
PIL	9.3 ± 0.2	8.9 ± 0.2	10.7 ± 0.2	11.1 ± 0.2	10.7 ± 0.2
	9–9.5; 10	8.6–9.3; 6	10.4–11.1; 10	10.8–11.5; 5	10.4–11.1; 22
BL	18.4 ± 0.3	17.4 ± 0.3	20.8 ± 0.5	21 ± 0.7	20.7 ± 0.3
	17.9–19; 10	17–17.9; 7	19.9–21.4; 9	20.1–21.7; 4	20.1–21.3; 22
UTL	9 ± 0.2	8.5 ± 0.2	10.2 ± 0.2	10.8 ± 0.2	10.3 ± 0.2
	8.8–9.2; 10	8.3–8.9; 7	9.9–10.6; 9	10.6–11; 5	10–10.7; 22
P^4^M^3^	5.5 ± 0.1	5.3 ± 0.1	6.3 ± 0.1	6.4 ± 0.1	6.1 ± 0.1
	5.3–5.6; 10	5.2–5.4; 7	6.2–6.5; 10	6.3–6.5; 5	6–6.3; 22
IOB	4.8 ± 0.1	4.8 ± 0.1	5.6 ± 0.5	5.4 ± 0.1	5.4 ± 0.1
	4.6–5; 10	4.7–5; 7	5.3–6.9; 11	5.3–5.5; 5	5.2–5.6; 22
DIF	3.9 ± 0.1	3.7 ± 0.1	4.3 ± 0.1	3.9 ± 0.1	3.7 ± 0.1
	3.7–4; 10	3.6–3.9; 7	4.2–4.5; 11	3.8–4; 5	3.6–3.9; 22
CB	10.7 ± 0.2	10.4 ± 0.3	11.7 ± 0.2	11.8 ± 0.3	11.4 ± 0.2
	10.2–11; 10	9.8–10.7; 7	11.5–11.9; 10	11.3–12.1; 4	11.2–11.7; 22
CH	6.3 ± 0.2	6.2 ± 0.2	6.8 ± 0.1	7 ± 0.2	7 ± 0.1
	6–6.5; 10	5.9–6.6; 7	6.7–7; 10	6.8–7.2; 4	6.8–7.5; 22
MB	6.2 ± 0.2	6 ± 0.1	7.1 ± 0.2	6.9 ± 0.1	6.6 ± 0.1
	5.7–6.5; 10	5.8–6.1; 7	6.9–7.4; 11	6.8–7; 5	6.3–6.8; 22
M^2^M^2^	5.9 ± 0.2	5.7 ± 0.1	7 ± 0.1	6.8 ± 0.2	6.3 ± 0.1
	5.6–6.1; 10	5.6–5.8; 7	6.9–7.1; 11	6.7–7.2; 5	6–6.6; 22
PPD	3.9 ± 0.1	3.9 ± 0.1	4.5 ± 0.1	4.6 ± 0.1	4.3 ± 0.1
	3.8–4; 10	3.8–4; 6	4.3–4.7; 11	4.5–4.7; 5	4.2–4.5; 22
BMF	3.6 ± 0.1	3.4 ± 0.2	3.7 ± 0.1	3.5 ± 0.2	3.5 ± 0.1
	3.5–3.8; 10	3.1–3.6; 7	3.5–3.9; 10	3.2–3.8; 4	3.2–3.7; 22
ML	11.6 ± 0.2	10.8 ± 0.2	12.8 ± 0.4	13.6 ± 0.3	13.2 ± 0.3
	11.3–11.9; 9	10.5–11; 7	12.3–13.4; 11	13.3–14.1; 5	12.5–13.7; 22
LTR	8.3 ± 0.2	7.8 ± 0.2	9.1 ± 0.3	9.8 ± 0.1	9.5 ± 0.2
	8–8.5; 9	7.7–8.1; 7	8.7–9.6; 11	9.7–10; 5	8.9–9.7; 22
HCP	4.9 ± 0.2	4.7 ± 0.2	5.9 ± 0.2	6.7 ± 0.1	5.6 ± 0.3
	4.5–5.1; 10	4.5–4.9; 7	5.7–6.1; 11	6.6–6.9; 5	4.6–6; 22
HCV	3.1 ± 0.1	2.9 ± 0.1	3.7 ± 0.1	3.8 ± 0.1	3.2 ± 0.1
	3–3.2; 10	2.7–3.1; 7	3.5–3.8; 11	3.7–3.9; 5	3.1–3.5; 22
HAC	3.8 ± 0.2	3.6 ± 0.1	4.5 ± 0.2	4.6 ± 0.1	4.3 ± 0.1
	3.7–4.1; 10	3.4–3.8; 7	4.2–4.8; 11	4.5–4.8; 5	4–4.5; 22

Comparative morphological data of another 62 *Soriculus* specimens including *S.nigrescens* (11), *S.nivatus* (23), *S.medogensis* (7) and *S.minor* (21) were obtained from Chen et al. (2023). Principal component analysis (PCA) was employed to evaluate the morphological variation amongst populations of *Soriculus*. The PCA was implemented in SPSS 19.0 (SPSS Inc., USA) and all craniodental measurements were first log_10_-transformed. The morphological measurements of *S.beibengensis* sp. nov. was compared with other *Soriculus* species. The terminologies for morphological descriptions followed Motokawa and Lin (2005) and Chen et al. (2023).

The total genomic DNA of five *S.beibengensis* sp. nov. specimens was extracted from muscle or liver using a DNA extraction kit (Qiagen DNeasy Blood and Tissue Kit, Germany). Two mitochondrial genes (complete cytochrome b, *Cyt b* and segment of *12S rRNA*, *12S*) and segments of three nuclear genes (apolipoprotein B, *APOB*; breast cancer 1, *BRCA1*; and recombination activating protein 2, *RAG2*) were amplified using primers and PCR conditions similar to Chen et al. (2023). The PCR products were sequenced in both directions using the BigDye Terminator Cycle Sequencing kit v. 3.1 (ThermoFisher Scientific, USA) on an ABI 3730xl sequencer. The DNA sequences were assembled using SeqMan (DNASTAR, Lasergene v.7.1). In addition, corresponding sequences of other *Soriculus* species were downloaded from the GenBank database. Corresponding sequences of several Soricidae genera were retrieved from the GenBank and used as outgroups. All sequences used in the study were aligned using MUSCLE (Edgar 2004), then checked manually. The uncorrected p–distance of *Cyt b* gene between the species pairs were calculated in MEGA-X (Kumar et al. 2018).

Three concatenated datasets were used for the phylogenetic analyses: (1) mitochondrial genes (mtDNA, *Cyt b* + *12S*, 1963 bp), (2) nuclear genes (nDNA, *APOB* + *RAG2* + *BRCA1*, 1974 bp) and (3) the mitochondrial and nuclear genes (mtDNA + nDNA, *Cyt b* + *12S* + *APOB* + *RAG2* + *BRCA1*, 3937 bp). Maximum-Likelihood (ML) and Bayesian Inference (BI) methods were used to reconstruct phylogenetic relationships. The ML phylogenies were inferred using IQ-TREE (Nguyen et al. 2015), with 10000 ultrafast bootstraps to estimate branch support. The BI phylogenies were inferred using MrBayes 3.2.6 (Ronquist et al. 2012) with two parallel runs and 10,000,000 generations, in which the initial 25% of sampled data were discarded as the burn-in. Before the phylogenetic inferences, the best-fit partitioning scheme and evolutionary models were selected using PartitionFinder v.2.1.1 (Lanfear et al. 2017) with greedy algorithm and BIC criterion (Suppl. material 1). The above analysis were performed in PhyloSuite (Zhang et al. 2020). The posterior probabilities (PP) ≥ 0.95 and ultrafast bootstrap value (UFBoot) ≥ 95 were considered as strong support (Huelsenbeck and Rannala 2004; Minh et al. 2013).

We estimated divergence time using BEAST v.2.6.7 (Bouckaert et al. 2014) on the concatenated nuclear gene dataset for which also the best partition schemes and evolutionary models were estimated using PartitionFinder v.2.1.1 (Lanfear et al. 2017) with greedy algorithm and BIC criterion (Suppl. material 1). Divergence dates were calibrated, based on three secondary calibrations following Chen et al. (2023): (1) the split between Crocidurinae and Soricinae is estimated at about 36 Ma (95% confidence interval [CI] = 28.6–44.0 Ma; Springer et al. (2018)). We established the prior using log-normal distribution with offset: 0, mean: 36, standard deviation: 0.135, which will specify a distribution centred at about 35.7 Ma and the 95% CI was 28.6–44.5 Ma. (2) The oldest Blarinellini was from the Early Middle Miocene (Harris 1998, Rzebik-Kowalska 1998) and the oldest Blarinini was in the Barstovian (Repenning 1967) at approximately 13.6–16.3 Ma. We established the prior for the divergence of Blarinellini-Blarinini using log-normal distribution with an offset of 15 Ma, mean of 0 and standard deviation of 0.98, which will specify a distribution centred at about 16.0 Ma and the 95% CI was 15.2–20.0 Ma. (3) The oldest fossil of Nectogalini was in the Late Miocene (MN10, 9.7–11.5 Ma; Fejfar and Sabol (2005)). We established the prior for the divergence of Nectogalini–Notiosoricini using log-normal distribution with an offset of 9.7 Ma, mean of 0 and standard deviation of 0.95, which will specify a distribution centred at about 10.7 Ma and the 95% CI was 9.91–14.5 Ma. The analyses were implemented with 100 million generations, sampled every 10,000 generations. Each analysis used a random starting tree, a log-normal relaxed clock model and a birth-death tree prior. Finally, the convergence of the parameters was assessed using Tracer 1.7 (Rambaut et al. 2018) and the tree was annotated using FigTree v.1.4.4.

## ﻿Results

### ﻿Morphological analyses

**Figure 2.**
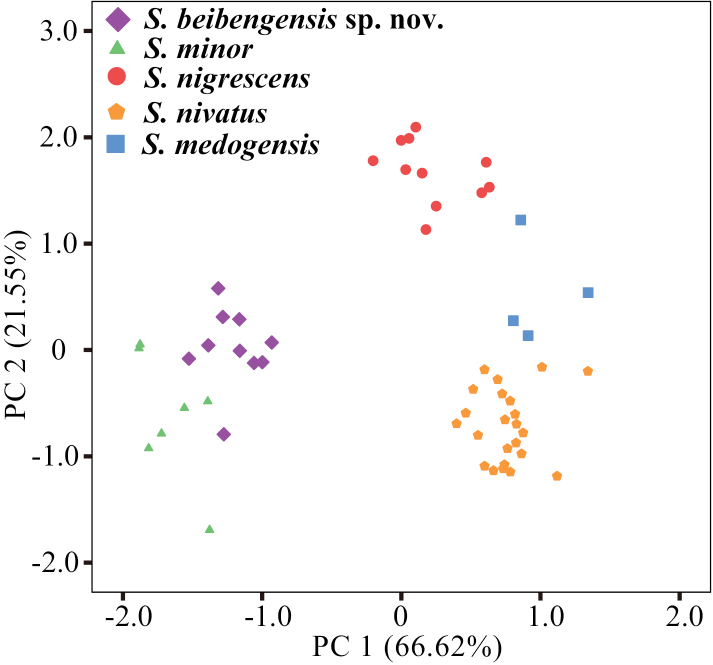
Results of principal component analysis (PCA) of *Soriculus*, based on the 18 log_10_-transformed craniodental measurements.

A summary of the external and cranial measurements of the five species under the genus *Soriculus* is given in Table 1. The *S.beibengensis* sp. nov. showed the highest morphometric similarity to *S.minor*, but is much smaller than *S.medogensis*, *S.nigrescens* and *S.nivatus*. The PCA, based on 55 intact skulls, produced two principal components with eigenvalues exceeding 1.0, which explained 88.17% of the variation (Table 2). The first principal component (PC1) explained 66.62% of the total variation and was strongly positively correlated with most of the variables (UTL, PIL, CIL, ML, LTR, BL, CH, P^4^M^3^, PPD, HAC, CB, HCP, IOB, M^2^M^2^, MB, DIF, BMF and HCV), indicating that it represented the overall skull size. The second principal component (PC2) explained 21.55% of the total variation and was strongly positively correlated with M^2^M^2^, MB, DIF, BMF and HCV (loading > 0.668), indicating that it represented the skull width. The plot of PC1 and PC2 (Fig. 2) showed that *S.beibengensis* sp. nov. is well-separated from other species, but positioned closer to *S.minor*. The two species plot on the negative axis of PC1, indicating the smaller skull in the genus. *S.beibengensis* sp. nov. is located in the upper right corner of *S.minor*, indicating it had a larger and wider skull compared to *S.minor*.

**Table 2.** Craniodental variation in *Soriculus*, based on principal component analysis (PCA). Character abbreviations are detailed in the “Material and methods”.

Character	PC1	PC2
UTL	0.973	0.168
PIL	0.972	0.166
CIL	0.960	0.178
ML	0.955	0.056
LTR	0.952	0.002
BL	0.950	0.202
CH	0.935	-0.022
P^4^M^3^	0.916	0.356
PPD	0.869	0.409
HAC	0.839	0.452
CB	0.839	0.458
HCP	0.826	0.398
IOB	0.743	0.371
M^2^M^2^	0.695	0.681
MB	0.674	0.670
DIF	0.100	0.932
BMF	-0.096	0.717
HCV	0.625	0.668
Eigenvalue	13.727	6.862
Variance explained	66.620	21.550

### ﻿Phylogenetic relationship

**Figure 3.**
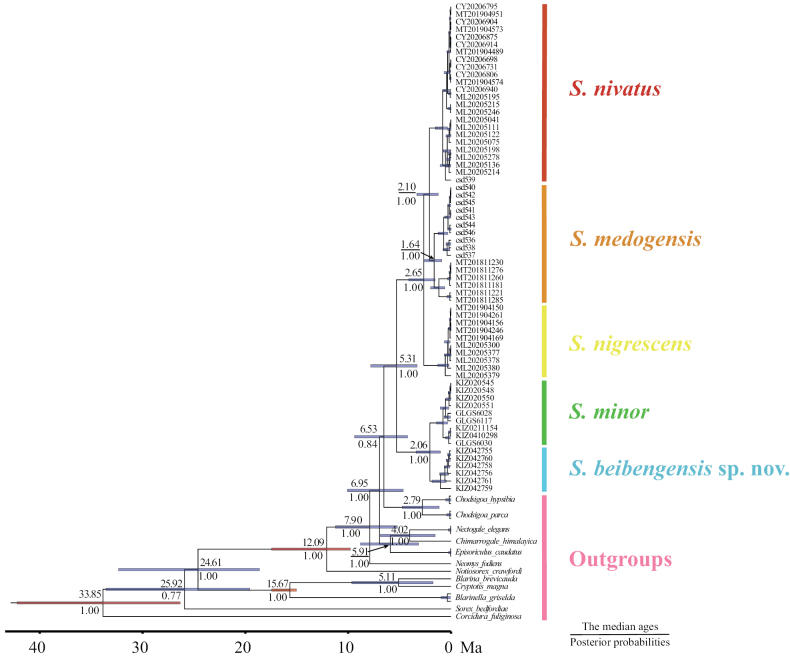
The phylogenetic tree inferred using Bayesian Inference (BI). The figure shows phylogenetic trees derived from **A** the mitochondrial genes **B** the nuclear genes, and **C** the mitochondrial and nuclear genes. The branch numbers indicate ultrafast bootstrap values (left) and BI posterior probabilities (right).

**Figure 4.**
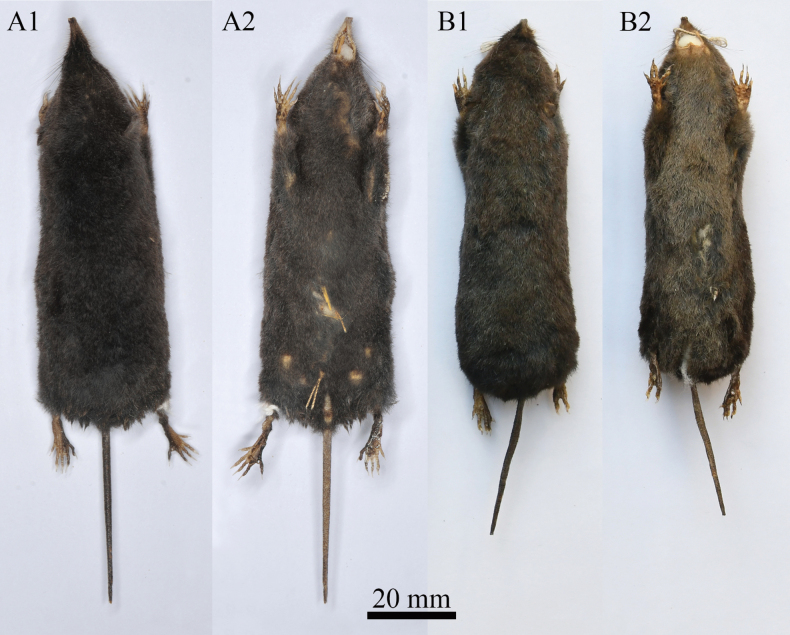
The divergence time of *Soriculus* species inferred from a time-calibrated phylogeny, based on the nuclear genes in BEAST v.2.6.7. The node numbers represent the median ages of the divergence times (upper) and posterior probabilities (below). The branch lengths represent divergence time, node bars indicate the 95% CI for each clade age and red bars indicate the calibration points.

In total, we obtained 3937 bp long sequences for five specimens of *S.beibengensis*, including 1140 bp *Cyt b*, 823 bp *12S*, 507 bp *APOB*, 693bp *RAG2* and 774 bp *BRCA1*. All new sequences have been deposited in the GenBank (Accession Numbers: PP213259–PP213263 and PP226949–PP226968, Suppl. material 2). The topologies of ML and BI trees of the three datasets were highly similar and only the BI gene trees are shown (Fig. 3). The phylogenetic trees generated from the datasets of nDNA and mtDNA + nDNA strongly supported the monophyly of *Soriculus* and clustered into two major clades: the first clade was composed of *S.minor* and *S.beibengensis* sp. nov. (Clade I) and the second clade was composed of *S.medogensis*, *S.nigrescens* and *S.nivatus* (Clade II) (PP = 1.00, UFboot ≥ 94). In contrast, the tree, based on concatenated mitochondrial genes, showed a closer phylogenetic relationship between Clade I and *Chimarrogalehimalyica*, *Nectogaleelegans* and *Neomysfodiens*, but this result was not significantly supported (PP = 0.79, UFboot = 62). A sister relationship of *S.beibengensis* and *S.minor* was strongly supported in all gene trees (PP = 1.00, UFboot = 100). The p–distance of the *Cyt b* gene between the *S.beibengensis* sp. nov. and other species ranged from 9.1% (with *S.minor*) to 15.9% (with *S.medogensis*) (Table 3). The results of divergence time estimates (Fig. 4) showed that *S.beibengensis* sp. nov. and *S.minor* diverged at the early Pleistocene (ca. 2.06 Ma, 95% CI = 1.05 – 3.40 Ma).

**Table 3.** The p–distance between *Soriculus* species, based on the *Cyt b* gene.

	*S.beibengensis* sp. nov.	*S.minor*	*S.nigrescens*	*S.medogensis*
*S.beibengensis* sp. nov.				
*S.minor*	0.091			
*S.nigrescens*	0.154	0.163		
*S.medogensis*	0.159	0.156	0.113	
*S.nivatus*	0.151	0.153	0.118	0.105

### ﻿Taxonomic account

#### ﻿ Soriculus beibengensis sp. nov.

Taxon classificationAnimaliaEulipotyphlaSoricidae

973C3E19-7051-5474-BDA2-5382A379A7FE

https://zoobank.org/516798F9-7726-47D0-9C81-F73436F24D96

##### Suggested common name.

Beibeng large-clawed shrew, 背崩大爪鼩鼱.

##### Type material.

***Holotype*.** KIZ042755, adult female, collected on 08 April 2023 by Mingjin Pu, at Beibeng Town, Medog County, southeast Tibet, China (29.219°N, 95.189°E, 1610 m a.s.l.). Dried skin, cleaned skull and muscle tissue are deposited in KIZ.

***Paratypes*.** Five specimens KIZ042756 (adult female), KIZ042757 (adult female), KIZ042758 (adult female), KIZ042759 (adult female), KIZ042760 (adult female). Collected from the type locality at Medog in April 2023 at elevations from 1500 m to 2125 m. All specimens are deposited in KIZ.

##### Specimens examination.

Four specimens KIZ042761 (adult female), KIZ042762 (adult male), KIZ042763 (adult female), KIZ042764 (adult female).

##### Etymology.

The specific Latin name *beibengensis* named for Beibeng, the type locality, with the Latin adjectival suffix –*ensis* means “belonging to”.

##### Diagnosis.

**Figure 5.**
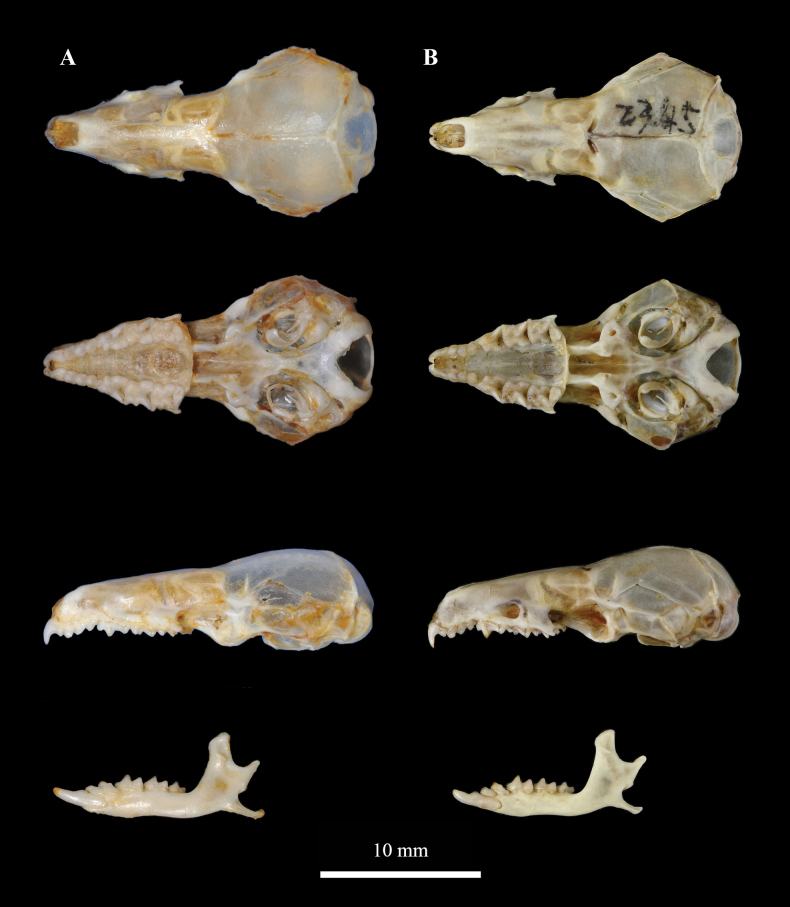
Dorsal and ventral view of the skin of **A***S.beibengensis* sp. nov. (KIZ042755) **B***S.minor* (KIZ020545).

**Figure 6.**
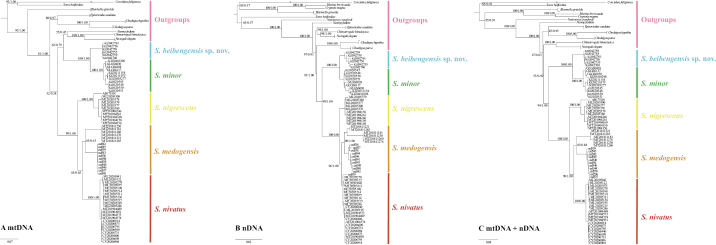
Dorsal, ventral and lateral views of the skull and mandibles of **A***S.beibengensis* sp. nov. (KIZ042755) **B***S.minor* (KIZ020545).

The new species is assigned to the genus *Soriculus*, based on the typically enlarged forefeet and claws (Fig. 5). Dark grey to black pelage; nearly similar ventral and dorsal pelage colour, similar to *S.minor* (Fig. 5); size (CIL: 20.8 ± 0.3 mm, 20.4–21.3 mm vs. 19.6 ± 0.4 mm, 19.2–20.2 mm; ML: 11.6 ± 0.2 mm, 11.3–11.9 mm vs. 10.8 ± 0.2 mm, 10.5–11.0 mm) larger than *S.minor*, but much smaller than *S.nivatus*, *S.nigrescens* and *S.medogensis*. The tail (40.6 ± 1.8 mm) is longer than *S.minor* (36.7 ± 4.1 mm), but shorter than *S.nivatus* (51.6 ± 2.7 mm), *S.nigrescens* (45.8 ± 3.2 mm) and *S.medogensis* (50.7 ± 3.6 mm). The TL/HBL (53%) is close to that of *S.minor* (52%) and *S.nigrescens* (52%), but smaller than *S.nivatus* (63%) and *S.medogensis* (60%). The nasal and rostrum are not clearly transitioned and seem to be stubby. The posterior process of the incisors widens, forming a narrow funnel-shaped channel between the processes. The basioccipital and basisphenoid are fused and narrowed, like a spade (Fig. 6).

##### Description.

Amongst five species of the genus *Soriculus*, *S.beibengensis* sp. nov. is the second smallest species. Its size is larger than *S.minor*, but smaller than *S.nivatus*, *S.nigrescens* and *S.medogensis* (Table 1). External morphology is similar to *S.minor*, with the dorsal pelage being dark grey to black and ventral pelage slightly paler. Tail is ambiguously bicoloured, dark above and pale below (Fig. 5). The tail is short (TL = 40.6 ± 1.8 mm, 38–44 mm), averages 53% of the head and body length and 8 of 10 specimens examined have a tail length more than 40 mm. The foreclaws are enlarged, similar to other *Soriculus* species. The back of hands and feet are covered by light brown to black hairs.

The skull is distinctly smaller than *S.nivatus*, *S.nigrescens* and *S.medogensis*, but larger than *S.minor*. Braincase is low and relatively flattened and the posterior of the skull is rounded. The sagittal and lambdoidal crest are well-developed and clear, the latter is especially prominent. The nasal and rostrum are not clearly transitioned and are stubby. The posterior process of incisor is widened, forming a narrowed funnel-shaped channel between the processes of adjacent teeth. The basioccipital and basisphenoid are fused and narrowed markedly in the middle region, forming a spade-like structure (Fig. 6).

The coronoid process is high and straight, with a concave anterior surface and a spatulate tip. The condyloid process has a single slender point and is angled upward at roughly 45 degrees, with the tip sitting below the coronoid process (Fig. 6). The angular process is long, straight and very thin, the tip slightly expands and bends upwards. The condyloid process is double-faceted, having two projections. The dental formula of the *S.beibengensis* sp. nov.is the same as the genus is: I 3/2, C 1/0, P 2/1, M 3/3 (×2) = 30. The apex of the first upper incisor is straight downwards, the tip of the first upper incisor is slightly pigmented with orange. There are four upper unicuspids (U^1^–U^4^); U^1^ is the highest, followed by U^2^, U^3^ and U^4^ is the smallest. M^1^ and M^2^ are similar in size, while M^3^ is reduced. The lower incisor (I_1_) is long, with only a low cusp and the tips are pigmented with orange. The lower unicuspid (U_1_) and P_4_ are crowded. M_1_ is larger than M_2_; M_3_ is the smallest.

##### Comparison.

Amongst species of the genus *Soriculus*, *S.beibengensis* sp. nov. is morphologically similar to its sister species, the *S.minor*. Both of them have a darker pelage and smaller size than other species. However, the new species can be distinguished from *S.minor* by multiple features. *S.beibengensis* sp. nov. is larger than *S.minor* for most of the external and craniomandibular measurements (Table 1). Especially, the skull of *S.beibengensis* sp. nov. is significantly longer than that of the *S.minor*, the measurements of CIL (20.4–21.3 mm vs. 19.2–20.2 mm) and ML (11.3–11.9 mm vs. 10.5–11.0 mm) between the two species do not overlap. The nasal and rostrum of *S.beibengensis* sp. nov. are not clearly transitioned and seem to be stubby, while *S.minor*, as well as the other species, has a clear transition of the nasal and rostrum (Fig. 6). The posterior process of incisors in *S.beibengensis* sp. nov. are widened, forming a narrowed funnel-shaped channel between the processes, whereas they are not widened in *S.minor* (Fig. 6).

*Soriculusbeibengensis* sp. nov. can be easily distinguished from *S.nigrescens*, *S.nivatus* and *S.medogensis* by its smaller size, the darker pelage colour and almost no pigmentation of the teeth (Fig. 5). Compared to *S.nivatus*, the measurements of CIL, PIL, BL, UTL, P^4^M^3^, IOB, CB, CH, PPD, ML and LTR of *S.beibengensis* sp. nov. are smaller, with no overlap and the teeth of *S.nivatus* are slender, appear to be the most delicate in the genus. Amongst *S.beibengensis* sp. nov., *S.nigrescens* and *S.medogensis*, the ranges of most of their external and craniodental measurements do not overlap (Table 1). The teeth of *S.beibengensis* sp. nov. are significantly smaller, but the teeth of *S.medogensis* are robust, with the broadest ramus region and the highest coronoid process in the genus. Compared to *S.nivatus* (TL/HBL = 63%) and *S.medogensis* (TL/HBL = 60%), the tail of *S.beibengensis* sp. nov. (TL/HBL = 53%) is shorter and the tail length of *S.nivatus* more than 46 mm, the tail length of *S.medogensis* usually more than 50 mm (6 of 7 (species measurements?)), while *S.beibengensis* sp. nov. less than 44 mm. The size arrangement of the unicuspids of *S.beibengensis* sp. nov. is similar to *S.minor*, U^1^ is the highest, followed by U^2^, U^3^ and U^4^ is the smallest, while other species usually have the largest U^2^, followed by U^1^, U^3^ and U^4^.

##### Distribution and habits.

*Soriculusbeibengensis* sp. nov. is known only from the type locality in Beibeng and Damu Town, Medog, Tibet, China at elevations from 1501 to 2123 m a.s.l. They were mainly distributed in mixed forest dominated by oak and a few individuals were distributed in conifer-broadleaf mixed forest.

## ﻿Discussion

The genus *Soriculus* is one of the least-studied small mammals. Owing to the limited studies, several species were not described until recently and it was considered a monotypic genus for a long time (Motokawa 2003; Burgin and He 2018). With a series of recent surveys conducted by Chinese scientists in the Himalayan Region, the diversity of the *Soriculus* has gradually been discovered with four species recognised (Chen et al. 2023). Herein, we described the fifth species, *S.beibengensis* sp. nov, collected in Medog, Tibet, China. In the genus *Soriculus*, *S.beibengensis* sp. nov. is morphologically similar to *S.minor*, both having darker pelage and smaller body size, but the former has a significantly larger body and skull size than the latter. Moreover, the phylogenetic tree showed that the sequences of the *S.beibengensis* sp. nov. clustered as a single clade, sister to *S.minor* and the *p*–distance between the two clades is up to 9.1%. According to the diagnostic and monophyletic species concept (Mayden 1997; Gutiérrez and Garbino 2018), we recognise *S.beibengensis* sp. nov. as a distinct species under the genus *Soriculus*.

As research has progressed, the evolutionary relationships amongst species of the genus *Soriculus* have become clearer. The genus is mainly split into two clades, representing two different evolutionary processes. The fossil evidence of Nectogalini shows that different taxa in this family migrated southwards from the late Miocene to the early Pleistocene (He et al. 2010). Therefore, *Soriculus* also likely migrated southwards under the influence of global cooling and desiccation and settled in the Gaoligong Mountains and Himalayas in southwest China. Our results showed that both clades of *Soriculus* have extant species distributed in Medog, indicating that the genus *Soriculus* entered Medog at least twice and adopted Medog as a key refuge. However, the current evidence is not sufficient to determine the specific dispersal history of the genus *Soriculus*. Deeper biological and geological surveys will become the key for analysing the evolutionary history of the genus.

### ﻿Key to the species of *Soriculus*

**Table:** 

1	Small; CIL < 22.0 mm, ML < 12.0 mm	**2**
–	Large; CIL > 22.0 mm, ML > 12.0 mm	**3**
2	CIL < 20.3 mm, ML < 11.2 mm	***S.minor***
–	CIL > 20.3 mm, ML > 11.2 mm	***S.beibengensis* sp. nov.**
3	Mandible well developed, the ramus region broader and coronoid process is high, HCP > 6.6 mm	***S.medogensis***
–	Mandible less developed, the ramus region is narrow and coronoid process is short, HCP < 6.1 mm	**4**
4	Maxillary region narrower, teeth are slender, M^2^M^2^ < 6.6 mm	***S.nivatus***
–	Maxillary region broader, teeth are robust, M^2^M^2^ > 6.8 mm	***S.nigrescens***

## Supplementary Material

XML Treatment for Soriculus beibengensis

## ﻿Additional information

### Author ORCIDs

Xiaoxin Pei ￼ https://orcid.org/0000-0001-7137-2600

Zhongzheng Chen ￼ https://orcid.org/0000-0003-3821-0145

Quan Li ￼ https://orcid.org/0000-0001-7536-5475

Laxman Khanal ￼ https://orcid.org/0000-0003-2411-3627

Xuelong Jiang ￼ https://orcid.org/0000-0003-2052-2490

### Data availability

All of the data that support the findings of this study are available in the main text or Supplementary Information.

## Supplementary materials

Supplementary material 1 The best-fit partitioning schemes and evolutionary models estimated using PartitionFinder Data type xlsx https://binary.pensoft.net/file/1001987This dataset is made available under the Open Database License (http://opendatacommons.org/licenses/odbl/1.0/). The Open Database License (ODbL) is a license agreement intended to allow users to freely share, modify, and use this Dataset while maintaining this same freedom for others, provided that the original source and author(s) are credited.Xiaoxin Pei, Zhongzheng Chen, Quan Li, Xueyou Li, Changzhe Pu, Kang Luo, Jing Luo, Mingjin Pu, Hongjiao Wang, Laxman Khanal, Xuelong Jiang

Supplementary material 2 Samples, sampling localities and DNA sequences used for molecular analyses Data type xlsx https://binary.pensoft.net/file/1001988This dataset is made available under the Open Database License (http://opendatacommons.org/licenses/odbl/1.0/). The Open Database License (ODbL) is a license agreement intended to allow users to freely share, modify, and use this Dataset while maintaining this same freedom for others, provided that the original source and author(s) are credited.Xiaoxin Pei, Zhongzheng Chen, Quan Li, Xueyou Li, Changzhe Pu, Kang Luo, Jing Luo, Mingjin Pu, Hongjiao Wang, Laxman Khanal, Xuelong Jiang
